# Prognostic clinical phenotypes associated with tumor stemness in the immune microenvironment of T-cell exhaustion for hepatocellular carcinoma

**DOI:** 10.1007/s12672-023-00819-8

**Published:** 2023-11-13

**Authors:** Genhao Zhang

**Affiliations:** Department of Blood Transfusion, Zhengzhou University First Affiliated Hospital, Zhengzhou, China

**Keywords:** T-cell exhaustion, Stemness, HCC, ICIs, Prognosis

## Abstract

T-cell exhaustion (TEX) and high heterogeneity of cancer stem cells (CSCs) are associated with progression, metastasis, and treatment resistance in hepatocellular carcinoma (HCC). Here, we aim to characterize TEX-stemness-related genes (TEXSRGs) and screen for HCC patients who are more sensitive to immunotherapy. The immune cell abundance identifier (ImmuCellAI) was utilized to precisely evaluate the abundance of TEX and screen TEX-related genes. The stemness index (mRNAsi) of samples was analyzed through the one-class logistic regression (OCLR) algorithm. Application of the non-negative matrix decomposition algorithm (NMF) for subtype identification of HCC samples. The different subtypes were assessed for differences in prognosis, tumor microenvironment (TME) landscape, and immunotherapy treatment response. Then, the TEXSRGS-score, which can accurately forecast the survival outcome of HCC patients, was built by LASSO-Cox and multivariate Cox regression, and experimentally validated for the most important TEXSRGs. We also analyzed the expression of TEXSRGs and the infiltration of CD8+ T cells in clinical samples using qRT-PCR and immunohistochemistry (IHC). Based on 146 TEXSRGs, we found two distinct clinical phenotypes with different TEX infiltration abundance, tumor stemness index, enrichment pathways, mutational landscape, and immune cell infiltration through the non-negative matrix decomposition algorithm (NMF), which were confirmed in the ICGC dataset. Utilizing eight TEXSRGs linked to clinical outcome, we created a TEXSRGs-score model to further improve the clinical applicability. Patients can be divided into two groups with substantial differences in the characteristics of immune cell infiltration, TEX infiltration abundance, and survival outcomes. The results of qRT-PCR and IHC analysis showed that PAFAH1B3, ZIC2, and ESR1 were differentially expressed in HCC and normal tissues and that patients with high TEXSRGs-scores had higher TEX infiltration abundance and tumor stemness gene expression. Regarding immunotherapy reaction and immune cell infiltration, patients with various TEXSRGs-score levels had various clinical traits. The outcome and immunotherapy efficacy of patients with low TEXSRGs-score was favorable. In conclusion, we identified two clinical subtypes with different prognoses, TEX infiltration abundance, tumor cell stemness index, and immunotherapy response based on TEXSRGs, and developed and validated a TEXSRGs-score capable of accurately predicting survival outcomes in HCC patients by comprehensive bioinformatics analysis. We believe that the TEXSRGs-score has prospective clinical relevance for prognostic assessment and may help physicians select prospective responders in preference to current immune checkpoint inhibitors (ICIs).

**Supplementary Information:**

The online version contains supplementary material available at 10.1007/s12672-023-00819-8.

## Introduction

T lymphocytes are essential for eliminating malignancies in the tumor microenvironment (TME). Naive T cells proliferate quickly and develop into effector T cell subsets in response to tumor antigens. Few activated T cells are transformed into memory T cells after antigen clearance, while the majority of effector T cells perish. These memory T cells quickly reawaken and carry out effector T cell tasks after being stimulated by tumor antigens once more. However, prolonged antigen stimulation causes effector T cells to gradually lose their ability to generate an immune response and memory phenotype, a process known as T-cell exhaustion (TEX) [1]. The expression of inhibitory receptors (IRs) such as PD1, TIM-3, LAG-3, and CTLA4 is consistently high in TEXs, in contrast to effector and memory T cells. These IRs not only indicate the degree of TEX but also adversely affect T cell function [2–5]: (1) via extracellular structural domains, IRs compete to isolate target receptors or ligands, (2) IRs can attenuate signals from activated receptors by modifying intracellular mediators, (3) IRs promote suppressive gene expression. It is yet unclear what part TEX plays in the anti-tumor defenses [6]. First off, TEX still generates inflammatory cytokines and granzymes that have anti-tumor actions, thus T cell depletion does not indicate that T cells are entirely depleted. Second, T-cell depletion protects against harm brought on by overactive immune responses as a reaction to persistent antigenic stimulation. Therefore, targeting these immune checkpoints can effectively alleviate TEX.

Cancer stem cells (CSCs), also referred to as tumor-initiating cells, are dormant, embryonic, self-renewing neoplastic cells that were first discovered in blood tumors and quickly found in solid cancers. Due to their part to tumor resilience to chemotherapy and radiation therapy as well as relapse, CSCs have garnered a lot of study interest [7]. Recent research has demonstrated that the inherent stem cell characteristics of CSCs and the external immunosuppressive TME are intricately linked [8]. On the one hand, signals from the TME, such as cytokines and growth factors, can trigger stem cell signals like EMT, Wnt, JAK/STAT, and NFB, promoting tumor development, metastasis, recurrence, and therapeutic resistance. However, these CSC innate signals can also trigger TME remodelings such as angiogenesis, collagen remodeling, and immune escape linked to PD1/PD-L1. These findings suggest that further research into the connection between CSC markers and TME may lead to novel approaches that can better the prognosis of hepatocellular carcinoma (HCC) patients undergoing chemotherapy.

Considering that both TEX and CSCs are strongly interlinked with poor survival outcomes in HCC patients and can affect the efficacy of immunotherapy [9, 10], there is a critical demand for efficient biomarkers to identify the tumor stemness and TEX to fully understand and eradicate suppressive TME, improve patient survival, and increase the effectiveness of anticancer therapy for HCC. In the present research, we found 146 genes, referred to as TEXSRGs, linked to both tumor cell stemness and TEX. Based on these genes, we found two distinct clinical phenotypes with different TEX infiltration abundance, tumor stemness index, enrichment pathways, and immune cell infiltration through the non-negative matrix decomposition algorithm (NMF), which were confirmed in the ICGC dataset. Utilizing eight TEXSRGs linked to clinical outcome, we created a TEXSRGs-score model to further improve the clinical applicability of this research. Regarding immunotherapy reaction and immune cell infiltration, patients with various TEXSRGs-score levels had various clinical traits. The outcome and immunotherapy efficacy of patients with low TEXSRGs-score was favorable. Overall, we investigated the regulatory mechanisms of tumor stemness and TEX further in the development of HCC and their function in the clinical evaluation of tailored immunotherapeutic response and disease prognosis.

## Methods

### Data obtainment

The 342 samples from The Cancer Genome Atlas-LIHC cohort (TCGA, https://xenabrowser.net/), 230 samples from the LIRI-JP cohort (ICGC, https://dcc.icgc.org/), and 50 clinical samples previously gathered were included in this study as displayed in Additional file 1: Table S1. Samples with insufficient clinicopathological characteristics or survival times of less than 30 days were disqualified. Additionally, 4419 genes related to tumor stemness were collected from the previous study [11]. The immune cell abundance identifier (ImmuCellAI, 2020.02) was utilized to precisely evaluate the abundance of TEX [12]. Genes between the high and low abundance of TEX were eliminated as TEX-related genes with cut-off criteria of p-value less than 0.05 and |logFC|≥ 0.5. Additional file 2: Table S2 displays the TEX-related genes, stemness-associated genes, and TEXSRGs. The stemness index (mRNAsi) of HCC samples was analyzed through the one-class logistic regression (OCLR) algorithm [13].

### Clinical phenotypes identification by the NMF algorithm

Two nonnegative matrices, W and H (i.e., A≈WH), were created from the expression of 146 TEXSRGs (Matrix A). The matrix A was factorized repeatedly, and the results were combined to provide consensus clustering of HCC samples. The cophenetic, dispersion and silhouette factors were used to determine the ideal number of clusters. Consensus clustering was carried out by using the brunet method and 200 nruns from the R package “NMF”. We further applied GSVA analysis with the R package ‘GSVA’ to evaluate the diversity in biological processes between distinct clinical phenotypes.

### Formulation and external validation of the TEXSRGs-related model

To identify genes with a substantial prognostic influence, each TEXSRGs underwent a single-variate Cox regression analysis using the "survival" software. Next, using LASSO-COX regression and multivariate Cox regression analysis, the prognostic model connected to TEXSRGs was created as previously reported [14]. TEXSRGs-score = ∑jCoefficient (Genej)*Expression (Genej). Using Kaplan–Meier survival curves and receiver operating characteristic (ROC) curves, we evaluated the validity of the TEXSRGs-related model in the TCGA and ICGC datasets.

### Estimation of immune cell infiltration and quantification of the effectiveness of immunotherapy

Calculation of immune and stromal scores based on the ESTIMATE algorithm to assess the level of infiltrating immune and stromal cells [15]. To further understand the variability, it is being done to infiltrate different types of immune cells in HCC TME using the CIBERSORT [16], xCELL [17], MCPcounter [18], and TIMER [19] databases. Online analysis was done using the Tumor Immune Dysfunction and Exclusion (TIDE) database (http://tide.dfci.harvard.edu/), which predicts the frequency with which immunotherapy for HCC patients will be beneficial. Also, we examined the changes in the expression of many immune checkpoint inhibitors (ICIs) in distinct TEXSRGs-score groups.

### Potential therapeutic drug prediction

Our TEXSRGs-related model specifically looked at the relationship between TEXSRGs-scores and 216 medications that were found in the CellMiner database [20]. A medicine was classified as tumor-sensitive if its Pearson correlation coefficient was more than 0.3 and its adjusted p-value was less than 0.001. The half-maximal inhibitory dosages (IC50) of the targeted medications were then predicted using the gene expression level to show the treatment sensitivity.

### Clinical samples using immunohistochemistry and qRT-PCR

The 50 sample tissues used in this study were obtained from HCC patients who underwent hepatic tissue resection from July 2022 to December 2022 at our hepatobiliary surgery department. All patients were diagnosed for the first time and had not received treatment. All patients signed an informed consent form. Using paraffin-embedded samples from HCC patients, IHC experiments utilizing anti-PAFAH1B3 (Proteintech, China), or anti-CD8 (Abcam, UK) antibodies were independently carried out. A Leica DM 2500 microscope was used to take photographs after secondary antibodies had been applied to the slides. Two different observers independently assessed and rated the immunostaining intensity of the specified proteins as previously described [21]. In addition, the comparative expression levels of genes in normal and tumor tissues were examined using qRT-PCR. The primer sequences of the genes are listed in Additional file 1: Table S3.

### Statistical analysis

R-4.2.1 produced the statistical analyses used in this investigation. For quantitative data, the Student’s t-test was used to evaluate the statistical significance of regularly distributed variables, and the Wilcoxon rank sum test was used to assess the statistical significance of non-normally distributed variables. The contingency tables were analyzed with the two-sided Fisher exact test. The R program “Survminer” was used to do a Kaplan–Meier survival analysis. The sample was divided into high and low TEXSRGs-score subgroups using the surv-cutpoint function in the ‘surv’ package. The prognosis classification performance of the TEXSRGs-related model was evaluated using receiver operating characteristic (ROC) curves, and the area under the curve (AUC) was determined using the ‘timeROC’ package. The Benjamini–Hochberg technique was used to correct for false discovery rate (FDR) for multiple hypothesis testing, and all comparisons were two-sided with an alpha level of 0.05.

## Results

### Identification and validation of TEXSRGs-related clinical phenotypes

**Fig. 1:**
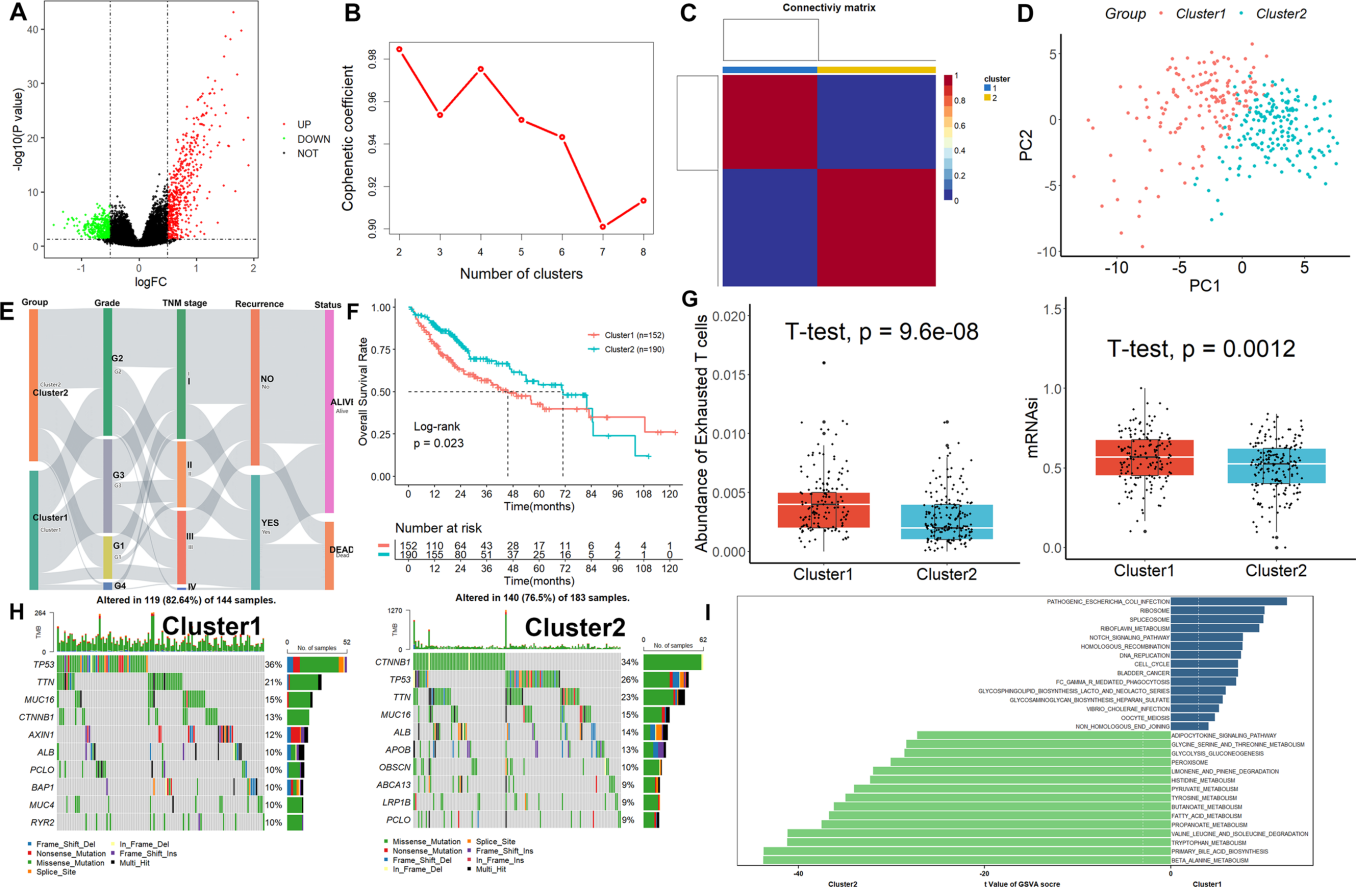
Identification of TEXSRGs-related clinical phenotypes in the TCGA cohort. **A** 892 genes were found as TEX-related genes. **B**, **C** When k = 2, the categorization of TEXSRG-related subgroups was the most reliable. **D** The PCA findings demonstrated that Cluster 2 patients could be easily differentiated from Cluster 1 patients. **E** There were substantial variations in Grade, TNM stage, recurrence rate, and patient survival status between the two clinical categories. **F** Compared to Cluster 1, HCC patients in Cluster 2 had superior mortality rates. **G** TEX and mRNAsi were more abundant in Cluster 1 HCC patients than in Cluster 2 patients. **H** Genetic mutation analysis. **I** GSVA analysis

The 342 HCC samples in the TCGA dataset were split into high and low categories based on the median value after the abundance of TEX was determined using ImmuCellA. As shown in Fig. 1A, 892 genes, including 447 upregulated genes and 491 downregulated genes, were found as TEX-related genes using cut-off parameters of p-value less than 0.05 and |logFC|≥ 0.5. These stemness-related genes were crossed with the TEX-related genes to create a total of 146 TEXSRGs, which were then used in the NMF clustering study. When k = 2, the categorization of TEXSRG-related subgroups was the most reliable (Fig. 1B, C). The PCA findings demonstrated that Cluster 2 patients could be easily differentiated from Cluster 1 patients (Fig. 1D). Furthermore, there were substantial variations in Grade, TNM stage, recurrence rate, and patient survival status between the two clinical categories (Fig. 1E). Compared to Cluster 1, HCC patients in Cluster 2 had superior mortality rates (Fig. 1F). Additionally, TEX and mRNAsi were more abundant in Cluster 1 HCC patients than in Cluster 2 patients (Fig. 1G). Genetic mutation research revealed that the two groups mutation rates were considerably different from one another (Fig. 1H). The sample with the highest mutation frequency in Cluster 1 was TP53, and the specific genes were AXIN1, BAP1, MUC4, and RYR2, while the sample with the highest mutation frequency in Cluster 2 was CTNNB1, and the specific genes were APOB, OBSCN, ABCA13, and LRP1B. Lastly, we discovered through GSVA analysis that Cluster 1 was primarily enriched in processes related to the cell cycle, whereas Cluster 2 was primarily enriched in processes related to metabolism (Fig. 1I).

**Fig. 2:**
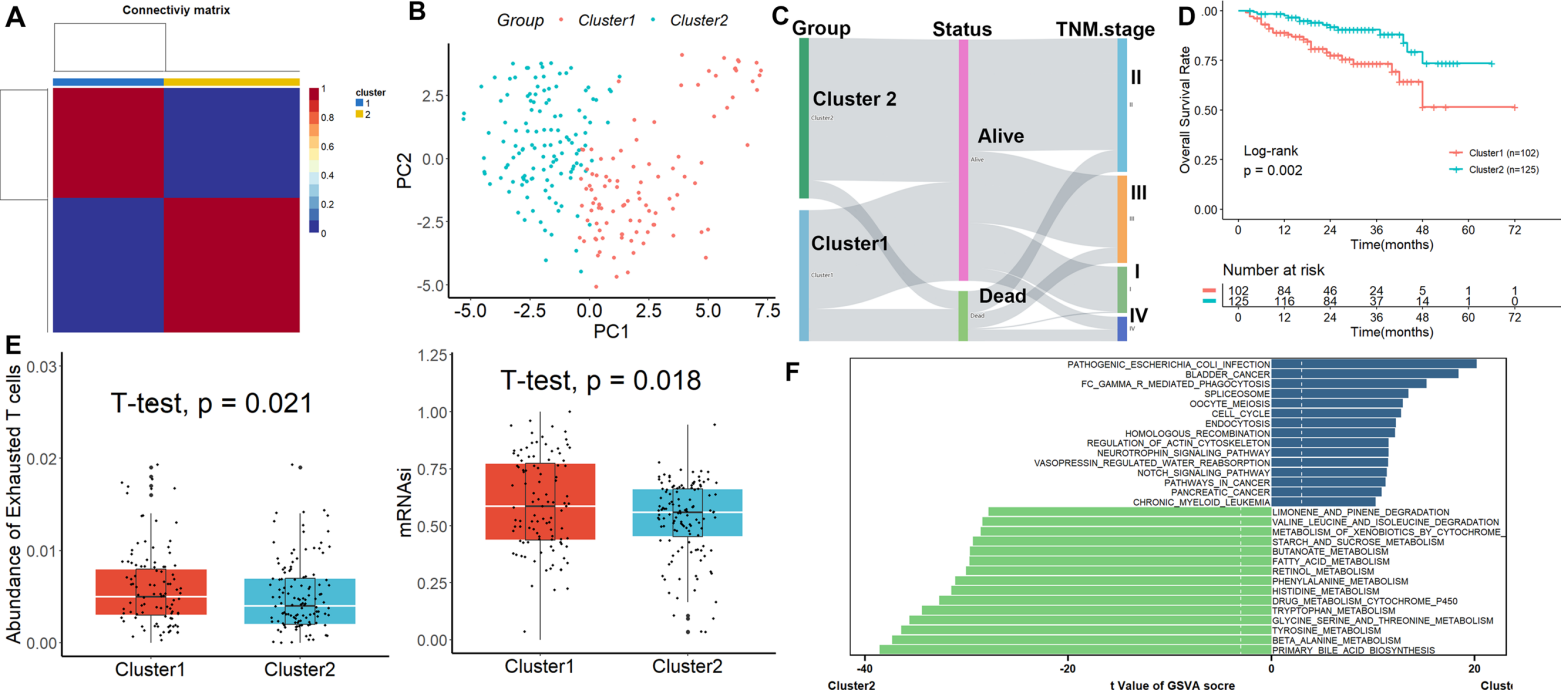
Validation of TEXSRGs-related clinical phenotypes in the ICGC cohort. **A** The HCC sample was divided into two subgroups. **B** Patients in Cluster 2 could be easily distinguished from those in Cluster 1. **C** There were also significant differences between the two clinical categories in terms of TNM staging and patient survival. **D** Mortality was higher in patients with HCC in Cluster 2 compared to Cluster 1. **E** TEX and mRNAsi were more abundant in HCC patients in cluster 1 than in cluster 2. **F** GSVA analysis

Subsequently, we validated the TEXSRG-related clinical phenotypes in the ICGC dataset. Similar to the TCGA dataset, the HCC sample was divided into two subgroups (Fig. 2A) and patients in Cluster 2 could be easily distinguished from those in Cluster 1 (Fig. 2B). There were also significant differences between the two clinical categories in terms of TNM staging and patient survival (Fig. 2C). Mortality was higher in patients with HCC in Cluster 2 compared to Cluster 1 (Fig. 2D). In addition, TEX and mRNAsi were more abundant in HCC patients in cluster 1 than in cluster 2 (Fig. 2E). Finally, we found by GSVA analysis that Cluster 1 was mainly enriched in cell cycle-related processes, while Cluster 2 was mainly enriched in metabolism-related processes (Fig. 2F).

### TME heterogeneity among different clinical phenotypes

**Fig. 3:**
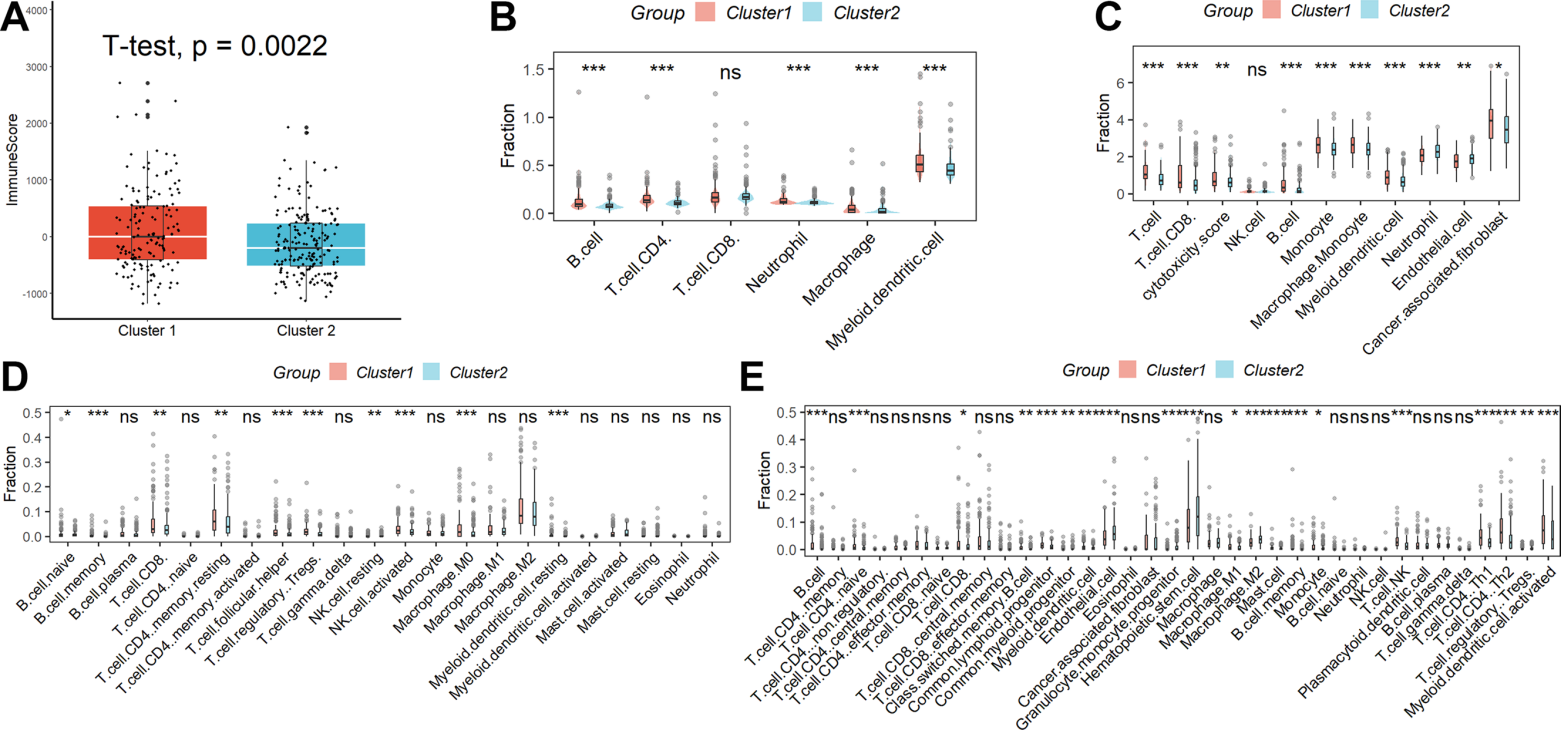
TME heterogeneity among different clinical phenotypes. **A** The results of the analysis are according to the ESTIMATE algorithm. Immunological profiles in a variety of immune-related cell types were characterized using TIMER (**B**), MCPcounter (**C**), CIBERSORT (**D**), and xCELL (**E**). *ns* not significant; *p < 0.05; **p < 0.01; ***p < 0.001

The results of the analysis according to the ESTIMATE algorithm showed that although there was no difference in stromal score and tumor purity between the two clinical subgroups, Cluster 1 had a significantly higher immune score than Cluster 2 (Fig. 3A). Subsequently, we further characterized their immunological profile in a variety of immune-related cell types using CIBERSORT, MCPcounter, TIMER, and xCELL. According to TIMER, samples in Cluster 1 had higher concentrations of B cells, CD4 T cells, neutrophils, macrophages, and myeloid dendritic cells than samples in Cluster 2, as shown in Fig. 3B. Figure 3C shows that samples in Cluster 1 in the MCPcounter database had higher abundance levels of T cells, CD8 T cells, B cells, monocytes, macrophage monocytes, neutrophils, endothelium cells, cancer-related fibroblasts, and myeloid dendritic cells than samples in Cluster 2. Comparing samples from Cluster 1 and Cluster 2, CIBERSORT found that Cluster 1 samples contained more naïve B cells, memory B cells, CD8 T cells, resting memory CD4 T cells, follicular helper T cells, Tregs, resting NK cells, activated NK cells, M0 macrophage, and resting myeloid dendritic cells (Fig. 3D). As determined by the xCELL database (Fig. 3E), samples in Cluster 1 had lower numbers of B cells, naïve CD4 T cells, CD8 T cells, class-switched memory B cells, common lymphoid progenitor, common myeloid progenitor, myeloid dendritic cells, M1 macrophage, memory B cell, mast cell, monocyte, NK T cells, Th1 CD4 T cell, Th2 CD4 T cell, Tregs, and activated myeloid dendritic cell when contrasted with those in Cluster 2. When the four aforementioned methods were merged, samples in Cluster 1 showed higher immune cell infiltrates in their TME. Considering the high level of immune cell infiltration but the poor prognosis in Cluster 1, we speculate that this may be related to TEX infiltration.

### The role of clinical phenotypes in predicting the efficacy of immunotherapy

**Fig. 4:**
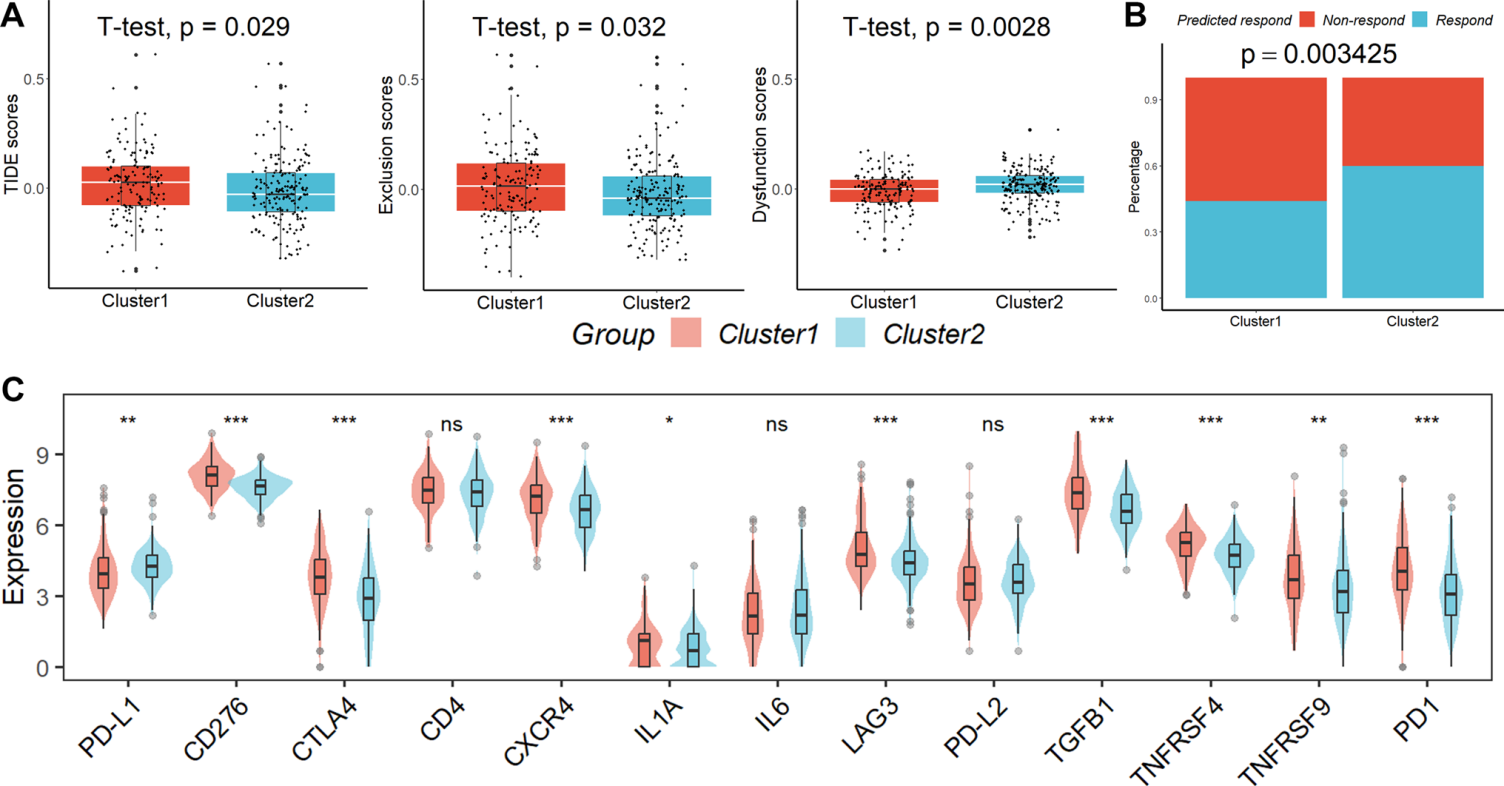
The role of clinical phenotypes in predicting the efficacy of immunotherapy. **A** TIDE analysis. **B** Cluster 1 had a reduced “response” proportion when compared to the anticipated immunotherapy response rate. **C** patients in cluster 1 had reduced levels of PD-L1 and higher levels of CD276, CTLA4, CXCR4, IL1A, LAG3, TGFB1, TNFRSF4, TNFRSF9, and PD1 compared to patients in cluster 2. *ns* not significant; *p < 0.05; **p < 0.01; ***p < 0.001

Even though the impacts of dysfunction scores were reversed, TIDE analysis revealed that cluster 1 had substantially greater TIDE and exclusion scores than cluster 2 (Fig. 4A). Cluster 1 had a reduced “response” proportion when compared to the anticipated immunotherapy response rate (Fig. 4B). Last but not least, we discovered that patients in cluster 1 had reduced levels of PD-L1 and higher levels of CD276, CTLA4, CXCR4, IL1A, LAG3, TGFB1, TNFRSF4, TNFRSF9, and PD1 compared to patients in cluster 2 (Fig. 4C).

### Identification and validation of TEXSRGs-related prognostic model

**Fig. 5:**
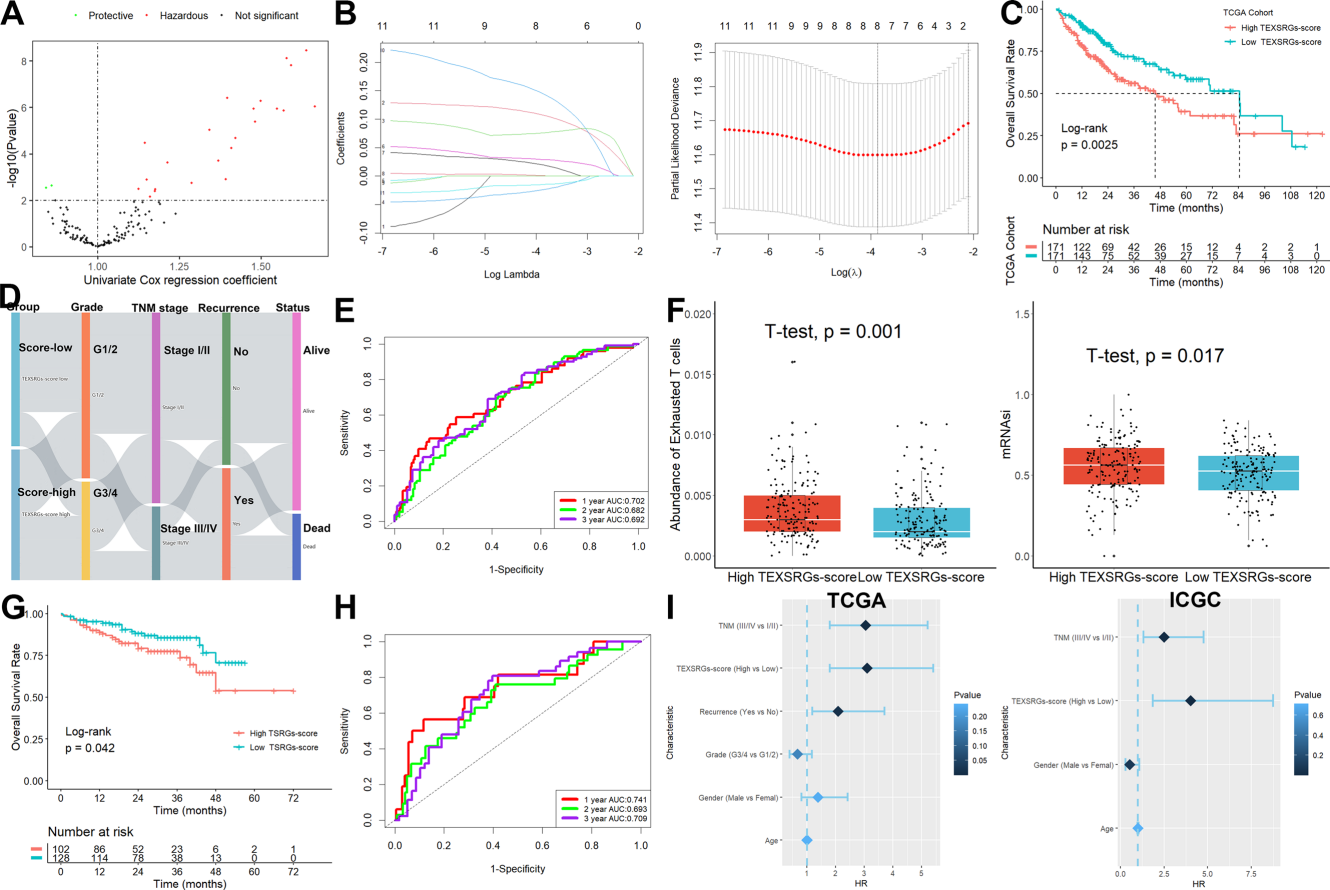
Identification and validation of TEXSRGs-related prognostic model. **A** Univariate Cox regression analysis. **B** LASSO Cox analysis. **C** Patients with high TEXSRGs-scores had considerably lower survival rates than patients with low TEXSRGs-scores. **D** The later grade, later TNM stage, recurrence, and survival status were associated with the high TEXSRGs-score. **E** The prognostic strength of the TEXSRG-related signature was assessed. **F** TEX and mRNAsi were more abundant in patients with high TEXSRGs-score than those with low TEXSRGs-score. **G** Patients with high TEXSRGs-scores had considerably lower OS than patients with low TEXSRGs-scores. **H** TEXSRGs-related signature performed well, with an AUC of 0.741, 0.693, and 0.709 for predicting the 1-, 2-, and 3-year survival of HCC patients, respectively. **I** The TEXSRGs-related signature was an independent influence on prognosis in both datasets

**Fig. 6:**
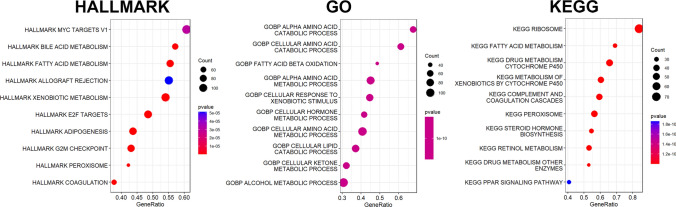
Identification of HALLMARK, GO, and KEGG enrichment between high- and low-TEXSRGs scores subgroups

**Fig. 7:**
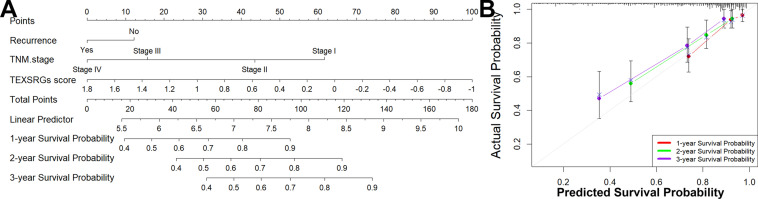
The predictive significance of the TEXSRGs-score was verified in the nomogram. **A** A nomogram model was built. **B** The likelihood of 1-, 2-, and 3-year survival rates predicted and actual overlap on the calibration curves showed good agreement

To further quantify the prognostic value of TEXSRGs in HCC, we did a univariate Cox regression analysis of TEXSRGs combined with survival status (Fig. 5A), and prognostically relevant TEXSRGs were obtained to be included in the LASSO Cox analysis (Fig. 5B). Eight genes (ZIC2, ESR1, PAFAH1B3, TNNT1, CDCA7, HMGA2, MYRIP, and FCER1G) were included to construct a TEXSRGs-score that could accurately predict the prognosis of HCC according to the following formula: TEXSRGs-score = (0.1298 × ZIC2) + (0.0349 × TNNT1) + (0.0371 × CDCA7) + (0.00487 × HMGA2) + (0.0691 × PAFAH1B3)—(0.01806 × MYRIP) + (0.1883 × FCER1G)—(0.0395 × ESR1). According to the median TEXSRGs-score, we determined the TEXSRGs-score for each sample and divided them into two groups: high and low. Patients with high TEXSRGs-scores had considerably lower survival rates than patients with low TEXSRGs-scores, according to the Kaplan–Meier curve (Fig. 5C). The later grade, later TNM stage, recurrence, and survival status were associated with the high TEXSRGs-score (Fig. 5D). By measuring the AUC, the prognostic strength of the TEXSRG-related signature was assessed. According to the findings, the TEXSRGs-related signature performed well, with an AUC of 0.702, 0.682, and 0.692 for predicting the 1-, 2-, and 3-year survival of HCC patients, respectively (Fig. 5E). Additionally, TEX and mRNAsi were more abundant in patients with high TEXSRGs-score than those with low TEXSRGs-score (Fig. 5F). Furthermore, we validated the TEXSRGs-related prognostic signature in the ICGC cohort. We found that patients with high TEXSRGs-scores had considerably lower OS than patients with low TEXSRGs-scores (Fig. 5G) and the TEXSRGs-related signature performed well, with an AUC of 0.741, 0.693, and 0.709 for predicting the 1-, 2-, and 3-year survival of HCC patients, respectively (Fig. 5H). The TEXSRGs-related signature was an independent influence on prognosis in both datasets (Fig. 5I). According to the GSEA results, the differential genes between the two groups were mainly enriched in biological processes related to energy metabolism (Fig. 6). Lastly, we developed a nomogram to predict the survival rates for HCC patients by integrating the TEXSRGs-score and TNM stages (Fig. 7A), which performed well (Fig. 7B).

### Role of TEXSRGs-score in predicting immunotherapy efficacy and target drug screening

**Fig. 8:**
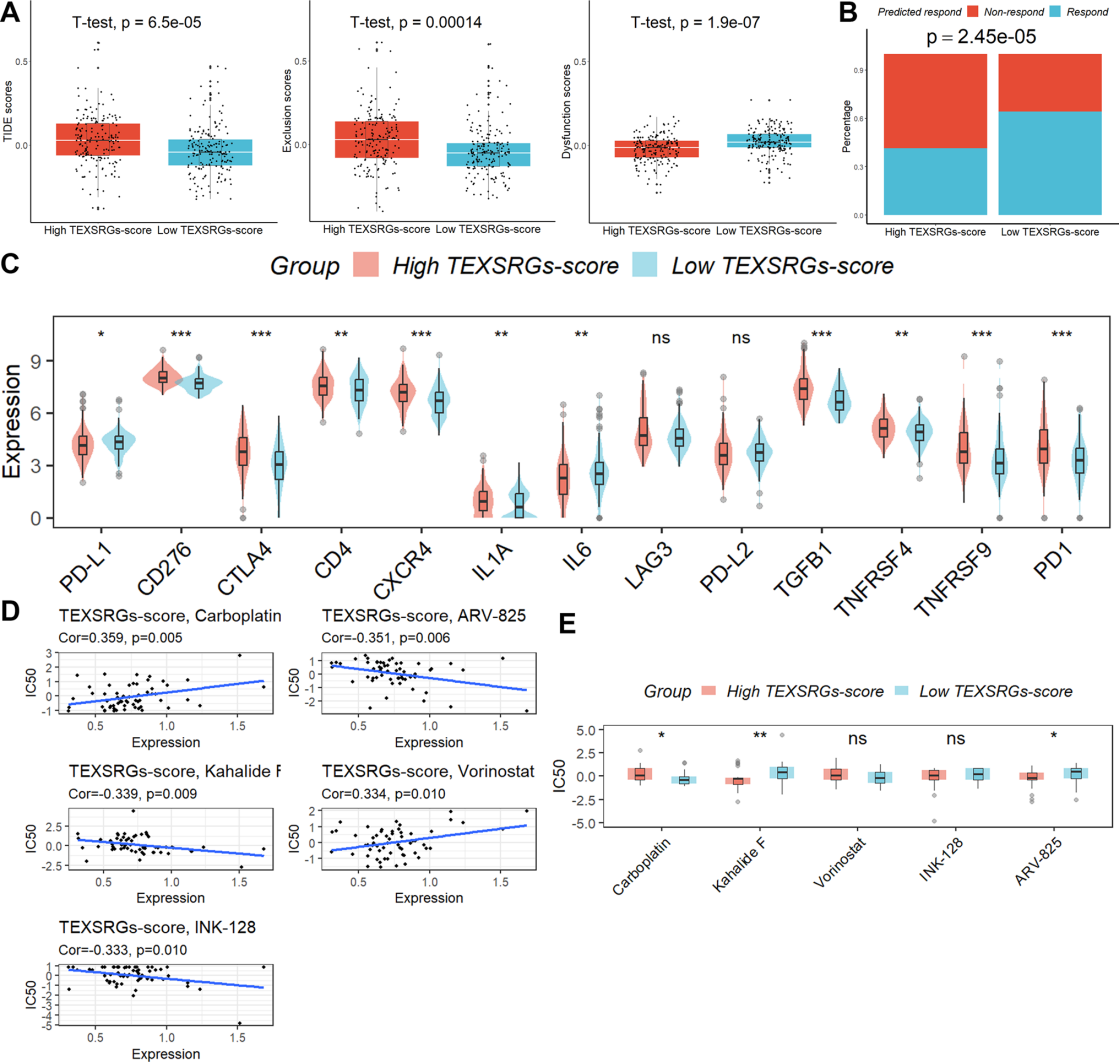
The role of TEXSRGs-score in predicting the efficacy of immunotherapy. **A** TIDE analysis. **B** Samples with high TEXSRGs-score had a reduced “response” proportion when compared to the anticipated immunotherapy response rate. **C** Difference analysis of ICIs genes. **D** The five tumor-sensitive medications. **E** Difference analysis of IC50 values. *ns* not significant; *p < 0.05; **p < 0.01; ***p < 0.001

Even though the impacts of dysfunction scores were reversed, TIDE analysis revealed that samples with high TEXSRGs-score had substantially greater TIDE and exclusion scores than those with low TEXSRGs-score (Fig. 8A). Samples with high TEXSRGs-score had a reduced “response” proportion when compared to the anticipated immunotherapy response rate (Fig. 8B). Moreover, patients with high TEXSRGs-score had reduced levels of PD-L1 and higher levels of CD276, CTLA4, CD4, CXCR4, IL1A, LAG3, TGFB1, TNFRSF4, TNFRSF9, and PD1 compared to patients with low TEXSRGs-score (Fig. 8C). Last but not least, we discovered five tumor-sensitive medications (Fig. 8D) and discovered that patients with higher TSRGs-scores had lower IC50 values for the medications kahalide F and ARV-825, indicating that they were more receptive to these medications (Fig. 8E).

### Relationship between TEXSRGs-score and TEX and tumor stemness in clinical samples

**Fig. 9:**
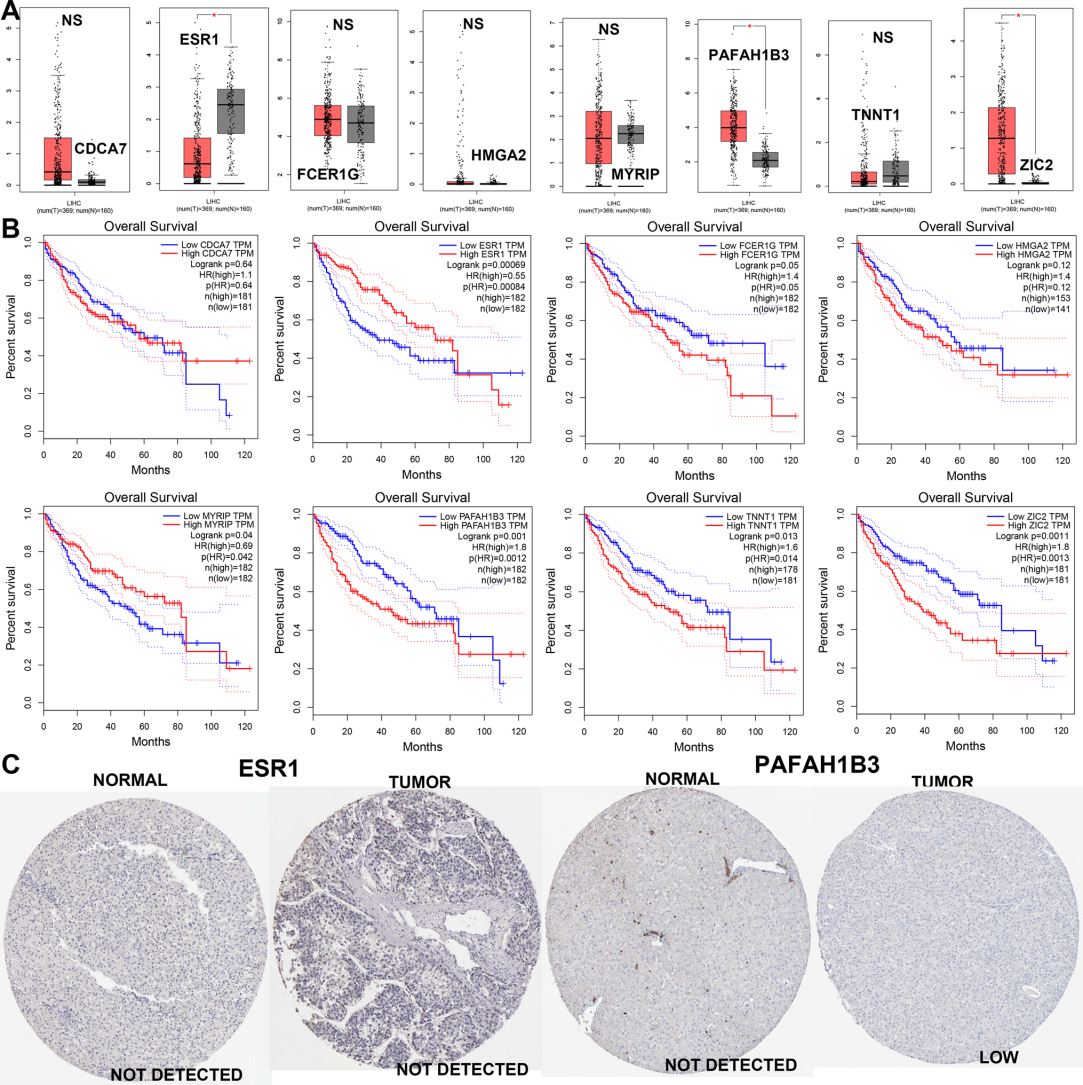
The expression of the eight TEXSRGs in normal and HCC tissues. **A** Expression levels and (**B**) prognostic values were explored in the GEPIA database. **C** Protein levels of ESR1 and PAFAH1B3 were explored in the HPA database. *ns* not significant; *p < 0.05

**Fig. 10:**
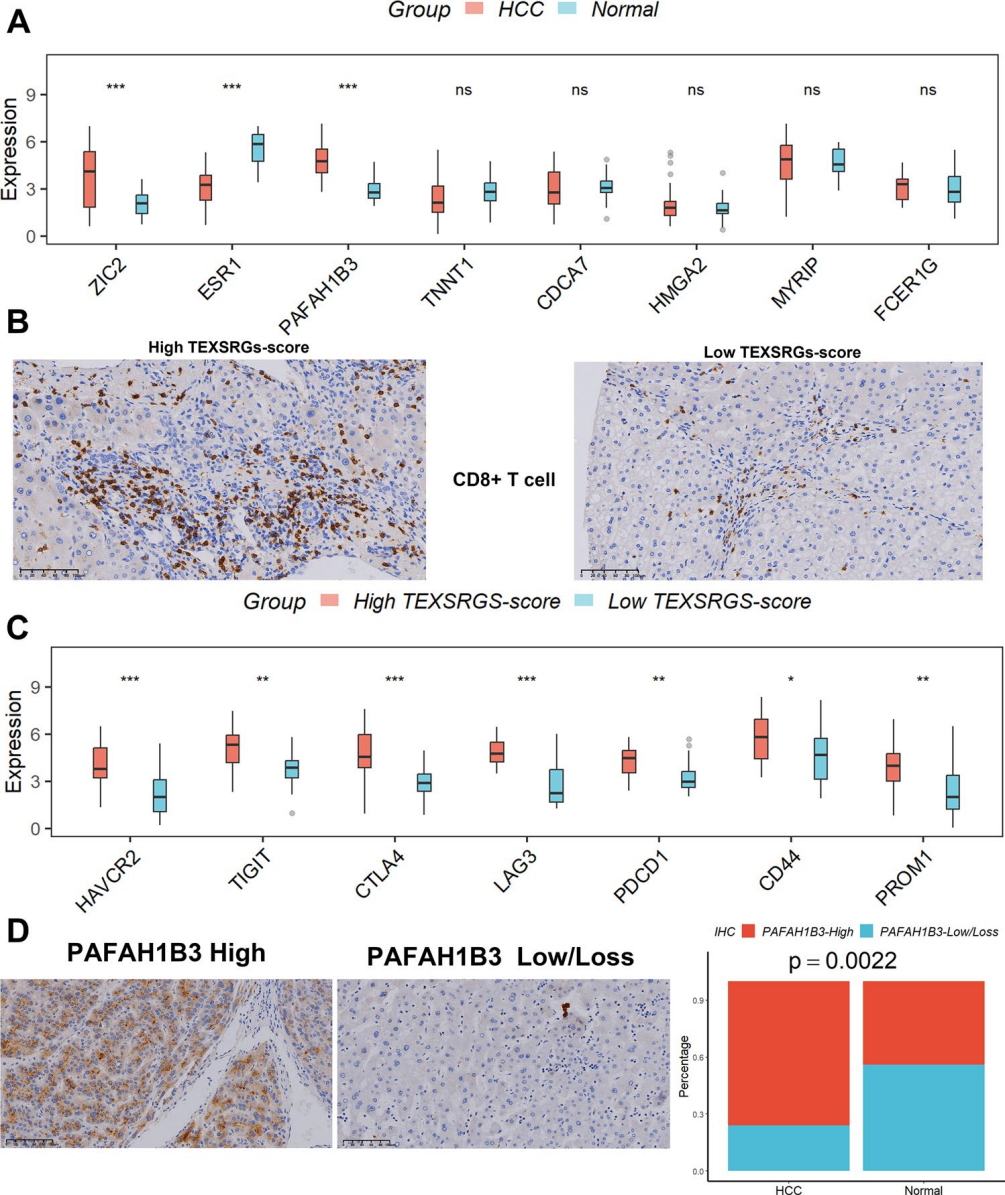
Relationship between TEXSRGs-score and TEX and tumor stemness in clinical samples. **A** The expression levels of the eight TEXSRGs in clinical samples were validated using qRT-PCR. **B** The percentage of infiltration of CD8 T cells was higher in the tumor tissues with high TEXSRGs-score than in those with low TEXSRGs-score. **C** The differences in expression levels of TEX marker genes (HAVCR2, TIGIT, CTLA4, LAG3, and PD1) and the putative tumor stem cell marker genes (CD44 and PROM1) between high and low TEXSRGs-score groups in clinical tumor samples were compared using qRT-PCR. **D** PAFAH1B3’s protein level. *ns* not significant; *p < 0.05; **p < 0.01; ***p < 0.001

According to the GEPIA database [22], increased expression of PAFAH1B3, ZIC2, and lower expression of ESR1 were found in HCC samples when compared to those in normal samples (Fig. 9A). We subsequently validated the expression levels of the eight TEXSRGs in clinical samples using qRT-PCR and found that the results were consistent with the GEPIA analysis (Fig. 10A). The expression of TEXSRGs in each sample was log-transformed, and the TEXSRGs-score of each sample was calculated by substituting the formula, and the clinical samples were divided into two groups of high and low TEXSRGs-score according to the median value. We then stained the tumor samples using IHC to detect the infiltration of CD8+ T cells in the tissues and found that the percentage of infiltration of CD8+ T cells was higher in the tumor tissues with high TEXSRGs-score than in those with low TEXSRGs-score (Fig. 10B). We also compared the differences in expression levels of TEX marker genes (HAVCR2, TIGIT, CTLA4, LAG3, and PD1) and the putative tumor stem cell marker genes (CD44 and PROM1) between high and low TEXSRGs-score groups in clinical tumor samples using qRT-PCR. We found that these genes were significantly upregulated in the high TEXSRGs-score group, suggesting that these samples may be in a state of high TEX infiltration and tumor stem cell activity (Fig. 10C). Furthermore, apart from CDCA7, FCER1G, and HMGA2, all genes were strongly associated with patient prognosis (Fig. 9B). Next, we focused on PAFAH1B3, ZIC2, and ESR1 because of their differential expression and prognostic potential. The human protein atlas (HPA) [23] database was performed to explore the three TEXSRGs’ protein expression in healthy and HCC tissues. Only PAFAH1B3 had differential expression among all of them (Fig. 9C). Lastly, we used immunohistochemistry to confirm its protein level and discovered that PAFAH1B3’s protein level was up-regulated in HCC tissues compared to normal tissues, which was compatible with the bioinformatics analysis discussed above (Fig. 10D).

## Discussion

TEX is a collection of cell subsets that operate differently from effector and memory T cells and is a major barrier to the advancement of effective cancer immunotherapy. The changed cytokines, altered epigenetic and transcriptional profiles, and altered metabolic rhythms are the most defining characteristics of TEX [1, 24]. PD-1, T-cell immunoglobulin and mucin structural domain 3 (TIM-3), lymphocyte activation gene 3 (LAG-3), CTLA4, and T-cell immunoreceptor with immunoglobulin and ITIM structural domains (TIGIT) are the most prevalent IRs. Monoclonal antibodies targeting these IRs can inhibit suppression of effector T cell exhaustion by blocking critical negative regulators of T cell function. However, immunotherapy’s untimely occurrence of immune-related adverse events has limited its clinical benefit [25]. Developing new strategies to target terminal TEX and restore immune function becomes the next windfall for improving immunotherapy efficacy.

CSCs are a relatively small subpopulation of cancer cells that can self-renew, proliferate indefinitely, differentiate multi-directionally and evade immune surveillance, and play an essential role in tumorigenesis. CSCs are also tightly associated with tumor invasion, metastasis, drug resistance, and recurrence after treatment. In addition to regulating reactive oxygen species (ROS) scavenging, epithelial-mesenchymal transition (EMT), and drug transport in TME, CSCs are also involved in the regulation of cellular autophagy and recycling [26, 27]. The high heterogeneity of CSCs, and their complex crosstalk with TME, hinder the clinical application of CSC-targeted therapies [11]. As a result of CSCs secreting TGF- and IL-4, CSCs become anti-apoptotic and CD8+ T cell-mediated anti-tumor immune responses are impaired [28]. Consequently, a thorough analysis of the highly heterogeneous CSCs, their adaptation, and their dynamic cross-talk with the TME ecosystem will make it easier to investigate particular, tailored therapeutic approaches for CSCs.

By using NMF analysis, we were able to identify two clinical subgroups in this research that differed in terms of clinicopathological features, mortality outcome, mutational landscape, and immune cell infiltration characteristics. This was done after we discovered the junction of TEX-related genes and tumor stemness-related genes. Notably, TEX infiltration and stemness index were more prevalent in Cluster 1. Additionally, we created a TEXSRGs-score to further measure the prognostic utility of these TEXSRGs in HCC. With the help of this score, patients can be divided into two groups with substantial differences in the characteristics of infiltration levels of immune cells, TEX infiltration abundance, and survival outcomes. On real clinical tissue samples, we validated the TEXSRGs-score in the end. We discovered that PAFAH1B3, ZIC2, and ESR1 were significantly different in tumor and normal tissues, which is consistent with the findings of the bioinformatic analysis. The infiltration of CD4 and CD8 T cells, as well as the levels of the TEX marker genes (HAVCR2, TIGIT, CTLA4, LAG3, and PD1) and tumor stem cell marker genes (CD44 and PROM1), were all greater in tissue samples with high TEXSRGs-score. These findings imply that the TEXSRGs-score we developed has high clinical practice applicability as well.

In addition, PAFAH1B3 protein was visibly elevated in HCC tumor tissue by IHC analysis, which is consistent with the previous report [29]. PAFAH1B3, a platelet-activating factor acetylhydrolase that causes platelet-activating factor inactivation by deacetylation, is involved in the regulation of cancer development. The expression of growth-inhibitory lipids is increased in breast cancer cells when PAFAH1B3 is blocked [30]. The proliferation, migration, and immunological infiltration of gastric cancer cells are all facilitated by PAFAH1B3 [31]. The effectiveness of treatment is enhanced by PAFAH1B3 blockade, which reduces the proliferation of HCC tumor cells [14, 32]. In this study, we found that PAFAH1B3 is not only linked with TEX infiltration but also involved in the regulation of HCC progression as a tumor stemness-related gene. In future work, we will investigate the mechanism by which PAFAH1B3 promotes HCC progression by influencing TEX and CSCs infiltration through more in vivo and in vitro experiments.

Finally, we also identified five tumor-sensitive drugs targeting TEXSRGs-score through the CellMiner database. Of these, patients with higher TEXSRGs-score had lower IC50 values for the drugs kahalide F (KF) and ARV-825, suggesting that these two drugs may be more effective in inhibiting disease progression in patients with higher TEXSRGs-score. KF is a new anti-cancer drug of marine origin with good safety and anti-tumor activity and stable metabolism in patients with advanced tumors [33]. KF promotes apoptosis by inducing oncosis and has been validated in several clinical trials and laboratory studies [34–37]. ARV-825 is a PROTAC bromodomain inhibitor that exerts anti-tumour activity by inhibiting the expression of MYCN or c-Myc [38–40]. Unfortunately, few studies have reported on the use of KF and ARV-825 in the antitumor treatment of HCC and it is worthwhile to recruit patients in future work to explore the efficacy and specific mechanisms of these two drugs in the antitumor treatment of HCC.

In contrast to earlier research, our work finds a clinical subtype of HCC with a high percentage of TEX and CSC infiltration and creates a TEXSRGs-score that reliably predicts patient prognosis and the effectiveness of immunotherapy. Our study does, however, have certain flaws. To confirm the prognostic ability of the TEXSRGs-score, external clinical data from multicenter large-scale HCC patient data with comprehensive follow-up information are first required. More in vivo and in vitro functional research is required to look into the biological effects of TEXSRGs on the infiltration of TEX and CSCs as well as TME landscapes. Finally, the true correlation between the TEXSRGs-score and immunotherapy response has to be evaluated in future immunotherapy cohorts due to the existing dearth of publically available transcriptome data on HCC patients receiving immunotherapy with ICIs.

## Conclusion

In conclusion, by unsupervised clustering of TEXSRGs, two clinical subtypes with different prognoses, TEX infiltration abundance, tumor cell stemness index, and immunotherapy response were systematically identified for the first time. A TEXSRGs-score that can precisely predict survival outcomes in HCC patients was then developed and validated. We conclude that the TEXSRGs-score has prospective clinical importance for HCC patients undergoing prognostic evaluation. This information may aid physicians in prioritizing the use of current ICIs by assisting them in selecting prospective responders.

### Supplementary Information

**Additional file 1: Table S1.** Clinical characteristics of HCC patients involved in the study. **Table S3**. The sequences of the qRT-PCR primers used in this study

**Additional file 2: Table S3.** The genes related to stemness and Exhausted T cells.

## Data Availability

The datasets used and/or analyzed during the current study are available from the corresponding author upon reasonable request.

## Abbreviations

TME: Tumor microenvironment
TEX: T-cell exhaustion
IRs: Inhibitory receptors
CSCs: Cancer stem cells
HCC: Hepatocellular carcinoma
NMF: Non-negative matrix decomposition algorithm
TEXSRGs: T-cell exhaustion and stemness-related genes
ImmuCellAI: Immune cell abundance identifier
mRNAsi: Stemness index
OCLR: One-class logistic regression
ROC: Receiver operating characteristic
TIDE: Tumor Immune Dysfunction and Exclusion
ICIs: Immune checkpoint inhibitors
IC50: Half-maximal inhibitory dosages
AUC: Area under the curve
FDR: False discovery rate
TIM-3: T-cell immunoglobulin and mucin structural domain 3
LAG-3: Lymphocyte activation gene 3
TIGIT: T-cell immunoreceptor with immunoglobulin and ITIM structural domains
EMT: Epithelial-mesenchymal transition
ROS: Reactive oxygen species
KF: Kahalide F
