# Supplementary material for: Prognostic clinical phenotypes associated with tumor stemness in the immune microenvironment of T-cell exhaustion for hepatocellular carcinoma
Source: Discov Oncol. 2023 Nov 13;14:203. doi: 10.1007/s12672-023-00819-8 (PMC10643807; doi:10.1007/s12672-023-00819-8)
Supplement: Supplementary file 1 — Additional file 1: Table S1. Clinical characteristics of HCC patients involved in the study. Table S3. The sequences of the qRT-PCR primers used in this study [file 12672_2023_819_MOESM1_ESM.docx]

Table S1 Clinical characteristics of HCC patients involved in the study

|  | TCGA cohort  (N=342) | ICGC cohort  (N=227) |
| --- | --- | --- |
| Gender Male | 233 | 61 |
| Female | 109 | 166 |
| Age ≤60 years | 165 | 49 |
| >60 years | 177 | 178 |
| Grade G1/2 | 214 |  |
| G3/4 | 123 |  |
| unknown | 5 |  |
| TNM Stage I/II | 238 | 140 |
| III/IV | 83 | 87 |
| unknown | 21 | 0 |
| Vascular Invasion Yes | 101 |  |
| No | 187 |  |
| unknown | 54 |  |
| Recurrence With tumor | 122 |  |
| Tumor free | 153 |  |
| unknown | 67 |  |
| Cirrhosis With | 65 |  |
| Without | 134 |  |
| unknown | 143 |  |
| AFP value <=400 ng/ml | 199 |  |
| > 400ng/ml | 61 |  |
| NA | 82 |  |
| HBV or HCV Infection |  |  |
| Yes | 141 |  |
| No | 161 |  |
| unknown | 40 |  |

Table S3 The sequences of the qRT-PCR primers used in this study

| Gene | Forward primer | Reverse primer |
| --- | --- | --- |
| ZIC2 | GCGCAACTCCACAACCAGTA | TGCCGCATATAGCGGAAAAAG |
| ESR1 | GAAAGGTGGGATACGAAAAGACC | GCTGTTCTTCTTAGAGCGTTTGA |
| PAFAH1B3 | ACATCCGGCCCAAGATTGTG | GGGCTGTCGCTCATTCACC |
| TNNT1 | TGATCCCGCCAAAGATCCC | TCTTCCGCTGCTCGAAATGTA |
| CDCA7 | GGGTGGCGATGAAGTTTCCA | GGGGATGTCTTCCACGGAAC |
| HMGA2 | ACCCAGGGGAAGACCCAAA | CCTCTTGGCCGTTTTTCTCCA |
| MYRIP | ACCTTCCTCGTCAACACCAAG | GTAGAACCATTCCAGAGATTGGG |
| FCER1G | AGCAGTGGTCTTGCTCTTACT | TGCCTTTCGCACTTGGATCTT |
| HAVCR2 | CTGCTGCTACTACTTACAAGGTC | GCAGGGCAGATAGGCATTCT |
| TIGIT | TCTGCATCTATCACACCTACCC | CCACCACGATGACTGCTGT |
| CTLA4 | GCCCTGCACTCTCCTGTTTTT | GGTTGCCGCACAGACTTCA |
| LAG3 | GCGGGGACTTCTCGCTATG | GGCTCTGAGAGATCCTGGGG |
| PDCD1 | CCAGGATGGTTCTTAGACTCCC | TTTAGCACGAAGCTCTCCGAT |
| CD44 | CTGCCGCTTTGCAGGTGTA | CATTGTGGGCAAGGTGCTATT |
| PROM1 | AGTCGGAAACTGGCAGATAGC | GGTAGTGTTGTACTGGGCCAAT |
| β-ACTIN | CGTGGGCCGCCCTAGGCACCA | TTGGCTTAGGGTTCAGGGGGG |
